# Comparison of pancreatic fat content measured by different methods employing MR mDixon sequence

**DOI:** 10.1371/journal.pone.0260001

**Published:** 2021-11-22

**Authors:** Xiaoyang Li, Qiushi Yang, Hang Ye, Shuo Li, Yuzhu Wang, Wanjiang Yu

**Affiliations:** 1 Department of Radiology, Qingdao Municipal Hospital, The Third Affiliated Medical College of Qingdao University, Qingdao, Shandong, China; 2 Dalian Medical University, Dalian, Liaoning, China; 3 Weifang Medical College, Weifang, Shandong, China; McLean Hospital, UNITED STATES

## Abstract

**Objective:**

To compare the reliability of different methods for measuring fat content of pancreas by MR modified Dixon(mDixon) Sequence and accurately evaluate pancreatic fat in as simple a way as possible.

**Methods:**

This is a retrospective study, 64 patients were included in this study who underwent abdominal MR scan that contained the mDixon sequence from June 2019 to May 2020(Included 7 patients with type 2 diabetes and 4 patients with impaired glucose tolerance (IGT), they were admitted to hospital through the obesity clinic set up by endocrine department, all of them were initially diagnosed and untreated). All of the 64 patients were scanned in 3.0T MR (Philips Ingenia II) due to their condition, 10–34 slice pancreas images were obtained, which were different from each other. Three different methods of measurement were employed by two observers using Philips Intellispace Portal software: (1) All images (whole-pancreas) measurement, the whole-pancreatic fat fraction (wPFF) was calculated by software. (2) Interval slices measurement, that is half-pancreatic slices fat fraction (hPFF) measured in the same way, fat fraction obtained by the interlayer assay was calculated. (3) As usual, the fat content of pancreatic head, body and tail fat was measured respectively, and in order to improve credibility, we also measured head、 body and tail in every layer, and its average value was taken. The elapsed time of the above different measurement methods was recorded. Intra-group correlation coefficient (ICC) was used to analyze the consistency of the measured data within and between observers. T-tests and Friedman tests were applied to compare the difference of measured values among groups.

**Results:**

No matter in normal person or diabetic or IGT, hPFF has shown good stability (ICChPFF = 0.988), and there was no significant difference compared with wPFF. But the average fat percentage composition of head, body and tail were significantly different from wPFF and hPFF (P < 0.01). At the same time, compared with normal person, pancreatic fat content in IGT and diabetic patients showed progressive significance(P<0.05).

**Conclusion:**

The distribution of pancreatic fat is not uniform, the method of measuring half pancreas by interlayer data collection can reflect the fat content of the entire pancreas, this suggests that measuring 50% of the pancreas is sufficient, this method effectively saves time and effort without affecting the results, which may have a better clinical application prospect.

## Introduction

It has been reported that pancreatic dedifferentiation and reversible cell dysfunction caused by pancreatic fat deposition may be a potential mechanism for type 2 diabetes [1, 2]. The reason is that the islets are exposed to increased fatty acids, which affect the normal function of the pancreas and lead to diabetes. At the same time, Stevens et al. [3] found that acute weight loss in type 2 diabetes resulted in loss of pancreatic parenchymal fat and improved clinical symptoms, these studies highlighting the importance of accurate quantification of pancreatic fat content. Also, there have been fairly extensive studies on the measurement of pancreatic fat [4–9], the researchers in these studies agreed that MRI is currently the preferred method for noninvasive measurement of pancreatic fat, however, all of the above studies used the method of placing 3–5 small circular regions of interest (ROI) in the homogeneous signal position of the pancreas in only one layer and calculating the average value, when we use this measurement method, we find that the measurement results are unstable and inaccurate, and the applicability is poor. In imaging studies, how to set the region of interest to get the best results is a hot topic. So, this study aims to explore the differences between different measurement methods and find a simple and accurate method to assess the degree of pancreatic fat accumulation.

## Methods

### The research object

From June 2019 to May 2020, 64 patients (40 males and 24 females, average age 42 years, included 7 patients with type 2 diabetes and 4 patients with IGT) were enrolled in this study. Inclusion criteria for subjects:(1) Age ≥18 years old, except pregnant and lactating women;(2) Not diagnosed with any space-occupying lesions, acute infections and complications of pancreas or liver or other organs;(3) No history of alcoholism or drug addiction;(4) No combined cardiopulmonary function and liver and kidney dysfunction;(5) No contraindication of MRI examination. These patients were inpatients in Qingdao Municipal Hospital and were screened into this study in October 2020, after data collection, none of the authors obtained any private information from the patients, this study did not cause any problems to the subjects; and all the subjects underwent MRI examination according to the need of the disease, so there is no additional financial burden of this study. Finally, MRI examination is a non-invasive and non-radiological examination, and the addition of Dixon sequence to the routine examination sequence only extended the scan time by about 40 seconds, and all subjects were informed and orally informed with their informed consent (The Institutional Research Human or Animal Ethics Committee, Qingdao Municipal Hospital, 2021–66).

### Method of measurement

**Fig 1:**
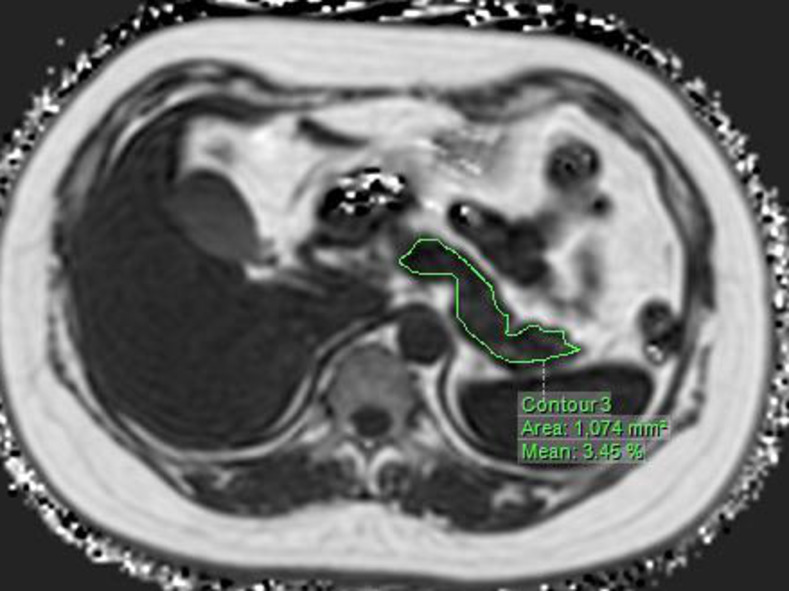
In the T1WI/FF sequence, the software can automatically calculate the fat content in the measured area by selecting the pancreas measurement range.

**Fig 2:**
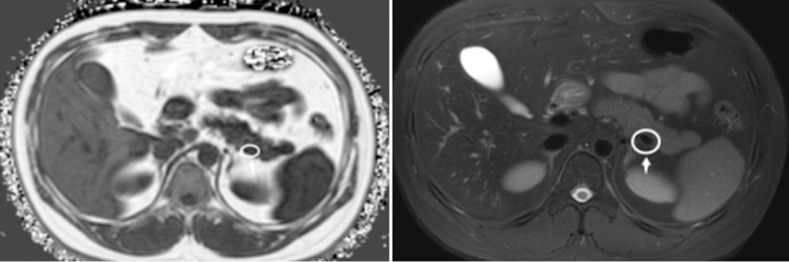
Note that tissues such as blood vessels also show low signal, and other sequences should be used to assist in the determination of pancreatic margins when only T1WI/FF sequence is difficult to distinguish.

**Fig 3:**
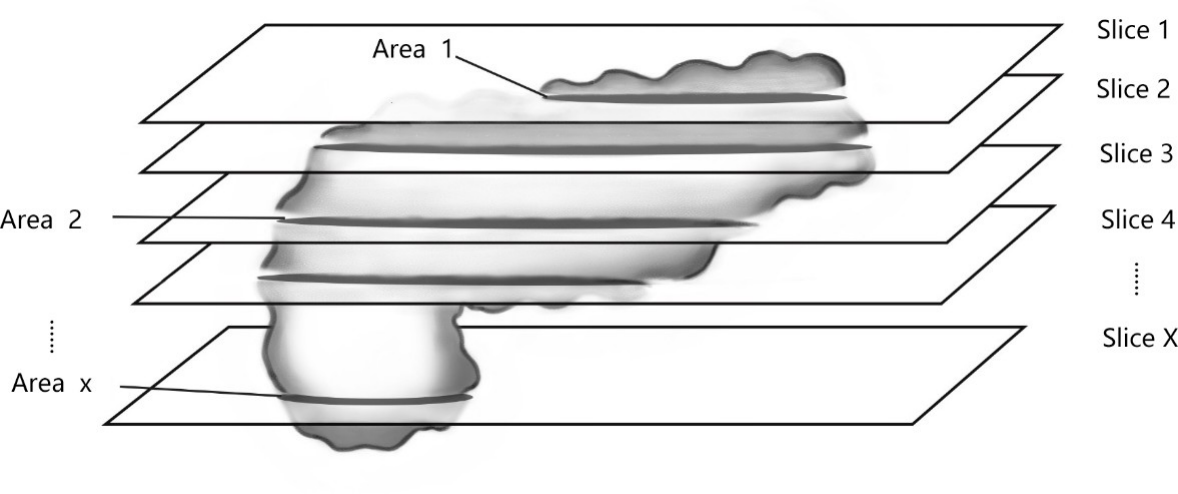
Calculation of pancreatic fat content by measuring all pancreatic layers (wPFF).

**Fig 4:**
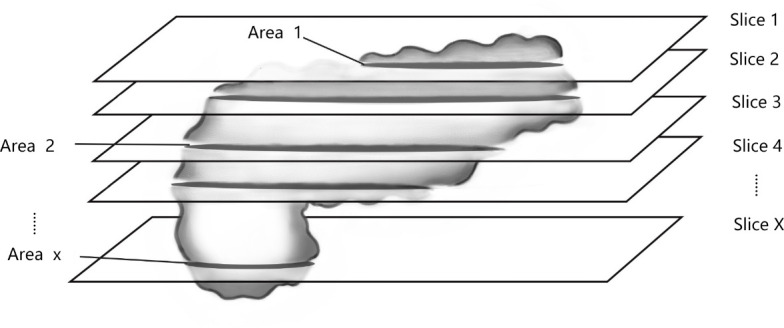
Interval selection measurement of pancreatic layer to calculate half-pancreatic fat content (hPFF).


1. MRI instrument and scanning method: Philips 3.0T Ingenia II MR instrument was used for magnetic resonance examination, and transverse axial mDixon water-lipid separation T1WI sequence scanning were performed, compared with Dixon sequence, the algorithm of mDixon sequence is more accurate. Imaging parameters: Imaging time:14 seconds; Rel.SNR:1; ACT TR:5.5ms,; TE1:0.92ms; Echo sequence length: 3; Overturn Angle: 3 degrees; Matrix: 152 x 113; Thick: 6 mm; Layer spacing: 3mm; FOV: 380 (RL) x285 (AP) x330 (FH) mm. The patient was supine on the examination bed and the scanning area was the upper abdomen.

2. Post-processing of the mDixon sequence images was performed using the Philips IntelliSpace Portal software with MRI workstations. Select the T1WI/FF sequence, and selecting the region of interest (ROI), the software can automatically display the area and fat content fraction (%) of the region(Figs 1 and 2).

3. Measurement of fat fraction: Under the double-blind principle, two observers measured all layers of the pancreas to calculate the whole-pancreatic fat content (wPFF) (Fig 3). And half of the total layers of the pancreas (hPFF) were measured in the same way(Fig 4).In addition, we placed several ROIs of 50mm² on the head, body and tail of the pancreas to measure the regional fat content in all layers. In the measurement process, the accompanying blood vessels and peripancreatic fat were strictly avoided. The calculation formula is equivalent to that described by Shingo Kato [10]:

$$\frac{\text{Area}1*\text{Mean}1+\text{Area}2*\text{Mean}2+\ldots +\text{Area}\;\text{X}*\text{Mean}\;\text{X}}{\text{Area}1+\text{Area}2+\ldots +\text{Area}\;\text{X}}$$

### Statistical analysis

All statistical analyses were performed using SPSS software (Version 25, SPSS Inc., Chicago, IL). The t-test was used to compare the data differences between the two groups, and the intra-group correlation coefficient (ICC) was used to analyze the consistency of the measured data within and between observers, and P < 0.05 was considered statistically significant.

## Results

### Consistency analysis

The consistency results of wPFF (%) data measured by two observers are shown in Table 1. The intra-observer ICC value is 0.997, indicating good consistency. The inter-observer ICC value was 0.996, showing good consistency. Meanwhile, the intra-observer ICC value of hPFF (%) obtained by interval measurement was 0.988, and the inter-observer ICC value was 0.988, which also showed good consistency. In contrast, the measurement methods used to calculate the average ROI for small areas are less consistent.

**Table 1:** Consistency of measured data between the two observers.

Item	N	Observer A¹	Observer A²	ICC (intra-observer)	Observer B	ICC (inter-observer)
wPFF (%)	64	8.38±6.28	8.39±6.17	0.997	8.34±6.15	0.996
hPFF (%)	64	8.36±6.18	8.24±6.10	0.988	8.27±6.07	0.988
Average (one layer, %)	64	6.33±5.42	5.72±5.36	0.891	6.08±5.06	0.890
Average (all layers, %)	64	7.36±5.99	6.91±5.67	0.889	7.27±5.59	0.896

(Data conform to normal distribution and are expressed as mean ± standard eviation).

In addition, the t-test results of wPFF (%) and hPFF (%) showed no significant difference (P > 0.05), indicating that a reliable fraction of pancreatic fat content could be obtained when we measured half of the layer, measuring 50% of the pancreas could reflects the fat content of the entire pancreas. The closer the measurement coverage is to the whole pancreas, the more reliable the measurement results will be. We also considered to further reduce the measurement layers, but the total pancreatic images of some subjects were less than 10 layers, so the further reduction of the measurement level was no longer applicable to these subjects, a certain measurement level should be maintained to obtain better applicability.

### Comparison of the whole fat content and local fat content of the pancreas

First, in the traditional way, we use small ROIs to measure head、body and tail in only one layer, the results of t-test showed that there were significant differences (P < 0.01) among the fat contents in the head, body and tail of the pancreas, the distribution of fat in the pancreas is uneven; extend the measurement range to all levels, the results is still the same.

**Fig 5:**
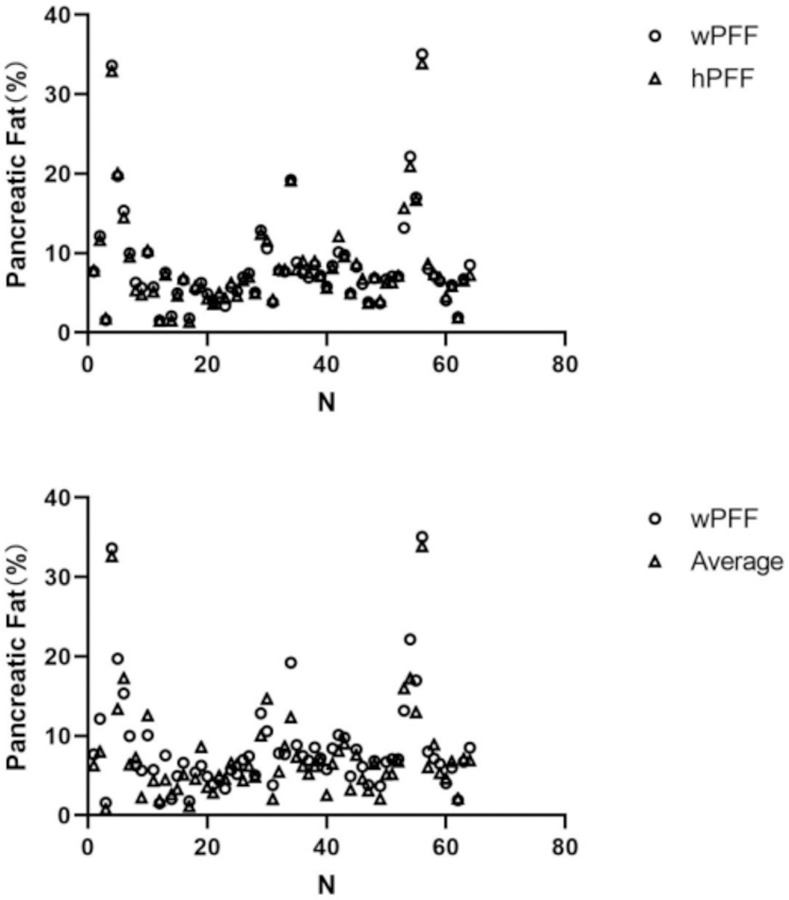
With wPFF as the evaluation standard, hPFF has a high degree of consistency, the average of head, body and tail are discrete from wPFF.

The whole pancreas measurement method is the most convincing of the many measurements and it has high repeatability, although the ICC value of the “Average of The Head、Body and Tail” is greater than 0.75, compared with wPFF (Fig 5), the mean value of pancreatic head, body and tail were significantly different (P < 0.01). The traditional method of measuring one layer of the pancreas using small ROI was not reliable, it is questionable whether the conclusions based on this method are reliable enough. In addition, measurements of the head, body and tail of the pancreas at all levels also did not show good stability.

### Relationship between pancreatic fat content and age, sex, IGT, type 2 diabetes mellitus

**Fig 6:**
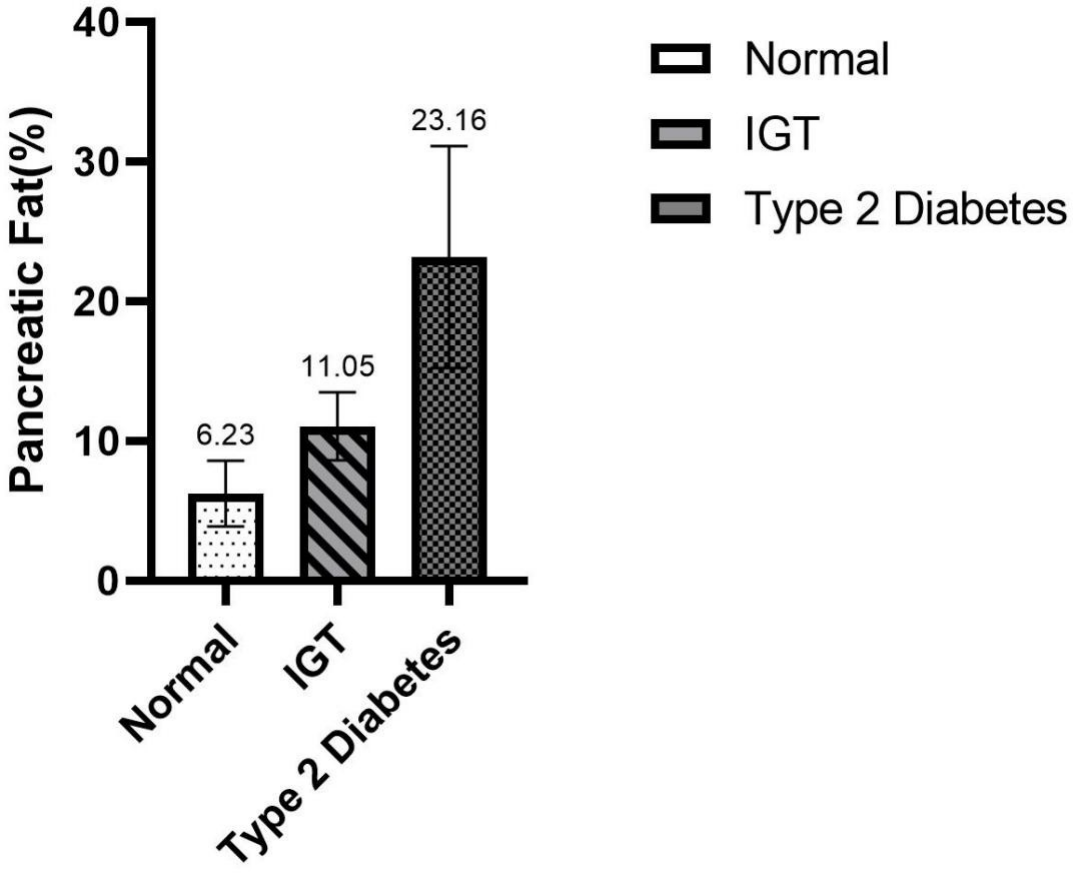
The fat content of pancreas in IGT group and diabetic group was higher than that in normal group, however, there was no significant characteristic difference on MR mDixon images of the pancreas, the difference in fat content can only be confirmed by measurement.

We obtained relevant diagnoses for all subjects by reviewing clinical data. The pancreas fat content of the normal group is 6.23±2.35, IGT group is 11.05±2.44, and the diabetic group is 23.16±7.94 (Fig 6). There was progressive significance among different groups(P<0.05), suggesting that pancreatic fat content can be used to assess the risk of diabetes. But when we did a statistical analysis of local pancreatic fat (head、body or tail) and diabetes, the results showed no clear correlation(P>0.05). In addition, there was no correlation between pancreas fat content and age and sex (P > 0.05).

### Comparison of time of different measurement methods

The mean time for the whole-plane pancreas measurement (wPFF) and interval measurement (hPFF) was 18.19±7.62mins and 6.57±4.93mins respectively. The t-test results showed that the duration of the two measurement methods was significantly different (P < 0.01). Kato’s measurement method has good reliability, but in practice, it still takes a lot of time and attention, so in clinical application, the interval measurement method can greatly save measurement time under the condition that wPFF can provide reliable results, solve the problem of time-consuming pancreas measurement, and has a good clinical application prospect.

## Discussion

Among all kinds of pancreatic fat measurement methods, most pathological biopsies can only be performed at autopsy and pancreatic surgery [11], it cannot be widely used in clinical practice because of its difficult operation, invasive and sampling error. Therefore, in recent years, studies have gradually tended to use the MR technique for the measurement of pancreatic fat.

In this study, first we find that when using a smaller ROI to measure pancreatic fat, the variation in the measurement results was largely dependent on the ROI location, this phenomenon also supports the conclusion of some studies that pancreatic fat is distributed unhomogeneously [6, 9, 10], Yidichen et al. [12] also observed the uneven distribution of pancreatic fat in live animals. These suggest that measurements based on small area ROI may not accurately represent the extent of pancreatic fat deposition, at the same time, the distribution of islet cells in the pancreas is not uniform [13], pancreatic fat may have different effects according to different accumulation sites. These findings also support the need for pancreatic fat quantification to be analyzed throughout the pancreas. In order to obtain more reliable measurement results, we tried to measure the overall fat content of the pancreas, and the results showed good consistency. The whole pancreas measurement method is no longer simply using small area ROI to calculate the average value, and can be regarded as a true reflection of pancreatic fat content.

In the process of reviewing the literature, we found that Shingokato and Ahmadal-Mahrabeh et al. [10, 14] also proposed to measure all levels of the pancreas and then calculate the overall fat content of the pancreas, which happened to agree with our idea. The approach taken by Shingokato et al. was to completely cover each layer of the pancreas with several ROIs of varying sizes. In another study, Ahmadal-Mahrabeh et al. used ImageJ mapping software to map the entire pancreas, and both methods showed stable measurements. The problem remains that it takes too long and is too cumbersome, so we tried to simplify a little bit, it turned out that interval measurements of the pancreas could yield stable and accurate results.

In addition, we found in our research that pancreatic fat is closely linked to diabetes, but this was only true when assessing overall fat in the pancreas, the relationship between local pancreatic fat and diabetes mellitus is haphazard. Moreover, pancreatic images from different populations did not have significant imaging features, we could not observe the sequence of fat accumulation in the pancreas, and there are very few images of the pancreas in diabetics showed atrophy of the pancreas and irregular increase in fat signaling, it’s not enough for the diagnoscian, so it is necessary to accurately measure pancreatic fat.

An important limitation of this study is the lack of pathological examination, and we were unable to directly compare the consistency of pancreatic fat content histologically and radiologically. Although MRI has a tissue resolution that is unmatched by other imaging methods, it is still unable to distinguish pancreatic intrafloular fat from interlobular fat. The precise location of fat deposition may require further analysis of the histological samples, but at present, MRI measurements of pancreatic fat content can be considered as a reliable indicator of pancreatic fat infiltration. Another regret is the lack of a large sample size.

In conclusion, different quantitative methods of pancreatic fat have great significance for the study of pancreatic fat deposition, how to accurately quantify pancreatic fat is worth further research. The quantitative method of whole pancreatic fat by MR mDixon technique is highly reliable and suitable for different populations. The interval measurement based on MR mDixon technique is stable and reliable, it is sufficient to reflect the fat accumulation of the entire pancreas. At the same time, the interval measurement method can save a lot of working time, so it has a better prospect of clinical application.

## Supporting information

S1 Data (XLSX)
